# Comparing Outcomes of Conventional Hemorrhoidectomy With Hemorrhoidectomy Using the Harmonic Scalpel

**DOI:** 10.7759/cureus.112426

**Published:** 2026-07-10

**Authors:** Gautham Vignesh, Ludia John, Sai Sravani Sure, Narayanan Cunnigaiper

**Affiliations:** 1 General Surgery, Sri Ramachandra Institute of Higher Education and Research, Chennai, IND; 2 Medicine, Sri Ramachandra Institute of Higher Education and Research, Chennai, IND

**Keywords:** harmonic scalpel, hemorrhoidectomy, hemorrhoids, milligan-morgan technique, postoperative pain

## Abstract

Background

Hemorrhoidal disease is one of the most common benign anorectal disorders encountered in surgical practice. Although various surgical modalities are available for grade III and IV hemorrhoids, Milligan-Morgan hemorrhoidectomy remains the gold standard treatment. However, postoperative pain is a major complication associated with this procedure. Therefore, hemorrhoidectomy using the harmonic scalpel (HS) has gained attention because of its potential to reduce tissue injury and postoperative pain.

Methods

This prospective single-center observational study included 112 patients with grade III and IV hemorrhoids who underwent hemorrhoidectomy using the conventional technique (n = 59) or the harmonic scalpel (n = 53) at a tertiary teaching hospital between December 2024 and May 2026. Approval was obtained from the Institutional Ethics Committee of Sri Ramachandra Institute of Higher Education and Research (approval number: CSP-MED/24/OCT/110/327), and written informed consent was obtained from all the participants. The study aimed to compare the efficacy of hemorrhoidectomy using the harmonic scalpel with the conventional technique in terms of perioperative outcomes. The primary outcomes included intraoperative bleeding, operating time, and postoperative pain, whereas secondary outcomes included the duration of hospital stay and postoperative urinary retention. Statistical analysis was performed, and a p-value of <0.05 was considered statistically significant.

Results

A significant reduction in operating time (<0.001), intraoperative blood loss (<0.001), postoperative pain (<0.001), postoperative urinary catheterization (0.018), and hospital stay (<0.001) was noted in the group undergoing hemorrhoidectomy using the harmonic scalpel compared with the conventional group.

Conclusion

Patients undergoing hemorrhoidectomy using the harmonic scalpel demonstrated a significant reduction in intra- and postoperative complications compared with the conventional group, suggesting greater efficacy in the management of grade III and IV hemorrhoids.

## Introduction

Hemorrhoidal disease, commonly referred to as piles, is characterized by the abnormal swelling or enlargement of the anal vascular cushions, resulting from a complex interaction between vascular dysregulation and the mechanical failure of supporting tissues, leading to the prolapse of anal cushions. Multiple factors, including constipation, excessive straining, extremely hard stools, and poor dietary habits, tend to contribute to the progression of the disease. Although the exact prevalence of the disease is unknown, many adults experience symptoms at least once between their 20s and 40s [1-4]. A prevalence of 16.6% was recorded in a large cross-sectional study of 194,620 healthy adults [5].

The management of hemorrhoids depends on the grade of the disease, symptom severity, and surgical expertise and ranges from simple lifestyle modifications to medical and surgical management. Milligan-Morgan hemorrhoidectomy is the gold standard treatment for grade III and IV hemorrhoids. Despite its efficacy, it is associated with significant postoperative pain and other complications, including reactionary or secondary hemorrhage, urinary retention, fecal impaction, flatus incontinence, delayed wound healing, anal stenosis, prolonged hospital stay, and delayed return to normal activities. Postoperative pain remains one of the most significant concerns following conventional hemorrhoidectomy and is a major determinant of patient satisfaction and recovery [6].

Efforts to reduce postoperative pain and complications have led to increasing interest in energy-based surgical devices such as the harmonic scalpel (HS), which uses ultrasonic energy for simultaneous tissue cutting and coagulation while limiting lateral thermal spread and tissue injury, which may reduce postoperative pain, promote faster wound healing, and decrease analgesic requirements following surgery [7].

This study aimed to compare the efficacy of hemorrhoidectomy using the HS with the conventional technique among patients with grade III and IV hemorrhoids, with respect to operating time, intraoperative blood loss, postoperative pain, the duration of hospital stay, postoperative urinary retention, postoperative bleeding, and postoperative urinary catheterization.

## Materials and methods

Study design and setting

This is a single-center prospective observational study. Approval was obtained from the Institutional Ethics Committee of Sri Ramachandra Institute of Higher Education and Research (approval number: CSP-MED/24/OCT/110/327), and written informed consent was obtained from all the participants. The study was conducted in the general surgery department of a teaching hospital between December 2024 and May 2026.

Participants and data collection

A sample size of 115 was calculated from the study conducted by Ahmed et al., which compared the efficacy of hemorrhoidectomy using the harmonic scalpel in treating grade III and IV hemorrhoids with the conventional technique [8].

A total of 112 patients who underwent hemorrhoidectomy at our hospital were included in the study. All patients aged 18 years or older with grade III or IV hemorrhoids confirmed by clinical examination and/or proctoscopy, along with those who had not responded to conservative management, were included in the study. Patients with a history of previous hemorrhoidectomy, coexisting anal conditions (fissures and fistulas), and significant comorbidities affecting surgical outcomes (e.g., uncontrolled diabetes and coagulopathy) and pregnant women were excluded from the study.

Proforma

A proforma was created to record the demographic details (age and sex), clinical details (grade and number of hemorrhoids), and intra- and postoperative findings (operating time, intraoperative blood loss {estimated by counting the number of blood-soaked mops and gauze pieces}, postoperative pain assessed using the visual analog scale {VAS}, the duration of hospital stay, and the need for postoperative urinary catheterization).

Intervention

Participants were allocated into two groups based on the surgical technique used: hemorrhoidectomy performed using conventional methods versus the HS. As this was a prospective observational study, treatment allocation was not randomized and was based primarily on the patient’s ability to afford the additional cost associated with the harmonic scalpel device. Accordingly, patients who were able to afford the cost underwent hemorrhoidectomy using the HS, whereas the others underwent hemorrhoidectomy using the conventional technique. Senior surgeons with a surgical experience of more than 10 years from different teams of the same department performed the procedures. Standard perioperative care protocols were followed in both groups.

Conventional hemorrhoidectomy

Under strict aseptic precautions, the patient is placed in the lithotomy position, and hemorrhoidal cushions are identified following the retraction of the anal canal. The hemorrhoidal mass is grasped with forceps and dissected from the underlying sphincter, and the vascular pedicle is ligated at its base. The hemorrhoidal mass is excised, and the wound is kept open for healing by secondary intention.

HS hemorrhoidectomy

The operative steps in the HS technique largely follow the principles of the conventional technique, with changes in the method of dissection and hemostasis. The patient is placed in the lithotomy or prone position, and the hemorrhoidal cushions are identified. The hemorrhoidal mass is grasped with forceps and dissected using the HS along the submucosal plane. The vascular pedicle is coagulated without the need for suture ligation, and the hemorrhoidal tissue is excised, leaving the wound open to heal by secondary intention.

Postoperative course

Postoperative pain was assessed twice daily using VAS and was managed using intravenous paracetamol (administered six-hourly) and ketorolac (administered twice daily). Tramadol was used as rescue analgesia. Patients were followed up within a week after the surgery for the assessment of the surgical site and associated complaints and complications.

Data analysis

Continuous variables were expressed as mean ± SD and compared using the independent sample t-test. Categorical variables were expressed as frequencies and percentages and analyzed using the chi-square test or Fisher’s exact test, as appropriate. For variables presented in categories (such as operative time and blood loss range), the chi-square test was used. A p-value of <0.05 was considered statistically significant.

## Results

A total of 112 patients participated in the study, with 59 undergoing conventional hemorrhoidectomy and 53 undergoing hemorrhoidectomy using the HS. The baseline characteristics of the patients were noted and are presented in Table 1.

**Table 1:** Baseline characteristics of the patients HS: harmonic scalpel

Parameter	HS (n = 53)		Conventional (n = 59)	
	n	%	n	%
Distribution of age (years)				
<30	7	13.2	10	16.9
31-40	15	28.3	13	22
41-50	16	30.2	17	28.8
51-60	9	17	10	16.9
>61	6	11.3	9	15.3
Comparison of gender				
Male	35	66	40	67.8
Female	18	34	19	32.2
Grade of hemorrhoids				
Grade III	38	71.7	48	81.4
Grade IV	15	28.3	11	18.6
Number of hemorrhoids removed				
One	10	18.9	19	32.2
Two	32	60.4	30	50.8
Three	11	20.8	10	16.9

There were no statistically significant differences in age, sex, grade, or number of hemorrhoids removed between the groups. However, most patients were in the 41-50-year age group, with a higher male predominance. A larger number of patients presented with grade III than grade IV.

The intraoperative outcomes, including operative time and intraoperative blood loss, were assessed for both groups of participants, as described in Table 2.

**Table 2:** Intraoperative outcomes *Statistically significant HS: harmonic scalpel

Parameter	HS (n = 53)		Conventional (n = 59)		P-value
	n	%	n	%	
Operating time (minutes)					
<10	22	41.5	2	3.4	<0.001*
11-20	22	41.5	19	32.2	
21-30	8	15.1	15	25.4	
31-40	0	0	14	23.7	
41-50	1	1.9	8	13.6	
51-60	0	0	1	1.7	
Intraoperative blood loss (mL)					
<10	24	45.3	0	0	<0.001*
11-20	21	39.6	8	13.6	
21-30	4	7.5	15	25.4	
31-40	1	1.9	6	10.2	
41-50	3	5.7	19	32.2	
51-60	0	0	11	18.6	

A significant reduction in operating time and intraoperative blood loss was observed in patients undergoing hemorrhoidectomy using the HS compared with the conventional group (p < 0.001).

Postoperative course

Postoperative pain (Table 3) and complications (Table 4) were assessed in both groups.

**Table 3:** Postoperative pain outcomes *Statistically significant HS, harmonic scalpel; POD, postoperative day

Parameter	HS (n = 53)	Conventional (n = 59)	P-value
	Mean ± SD	Mean ± SD	
POD 0	1.89 ± 0.70	3.17 ± 0.75	<0.001*
POD 1	1.17 ± 0.70	2.37 ± 0.81	
POD 2	0.55 ± 0.64	1.80 ± 0.92	

**Table 4:** Postoperative complications *Statistically significant HS: harmonic scalpel

Parameter	HS (n = 53)		Conventional (n = 59)		P-value
	n	%	n	%	
Need for urinary catheterization					
No	52	98.1	50	84.7	0.018*
Yes	1	1.9	9	15.3	
Postoperative bleeding					
No	52	98.1	57	96.6	1.000
Yes	1	1.9	2	3.4	
Duration of hospital stay (days)					
<3	47	88.7	13	22	<0.001*
4-6	6	11.3	45	76.3	
>7	0	0	1	1.7	

Postoperative pain was significantly lower in patients who underwent hemorrhoidectomy using the HS than in the conventional group (p < 0.001).

The need for urinary catheterization and the duration of hospital stay were lower in the patients undergoing hemorrhoidectomy using the HS than in the conventional group (p = 0.018 and p < 0.001, respectively). Although the rates of postoperative bleeding were lower among patients undergoing hemorrhoidectomy using HS, the difference was not statistically significant.

## Discussion

Hemorrhoids are caused by changes in both vascular and structural parts, leading to the enlargement and displacement of normal anal cushions. Most patients present with rectal bleeding, prolapse, and perianal itching. Untreated hemorrhoids can progress to profuse bleeding, thrombosis, strangulation, ulceration, suppuration, and gangrene; therefore, timely management becomes essential [9,10].

Simple conservative measures, such as dietary changes and lifestyle modifications, as well as medical management using oral and topical agents, are used to treat the disease in the early stages. When conservative management fails, more definitive treatments such as rubber band ligation, injection sclerotherapy, photocoagulation, laser hemorrhoidectomy, and suture ligation (Ligasure) for lower-grade disease (grades I and II), and surgical interventions such as hemorrhoidectomy using conventional technique (Milligan-Morgan method), the harmonic scalpel (HS), and stapled hemorrhoidopexy for higher grades (grades III and IV) are employed [11-13].

The Milligan-Morgan hemorrhoidectomy is the mainstay for treating grade III and IV hemorrhoids. Although efficacious in treating the disease, significant postoperative pain has known to be reported among patients undergoing the procedure. Severe postoperative pain is attributed to the rich nerve supply below the dentate line, causing high sensitivity to pain, along with other factors, including the number of hemorrhoids excised, local tissue injury, and inflammation. Managing postoperative pain becomes essential as it directly determines the speed of recovery, quality of life (QoL), and patient satisfaction [14].

In view of the increasing prevalence of postoperative pain following hemorrhoidectomy using the conventional technique, recent advancements have been made in energy-based devices such as the HS. The HS uses ultrasonic energy for simultaneous cutting and tissue coagulation, leading to minimal lateral thermal injury, reduced tissue necrosis, less sphincter irritation, and lower inflammatory response, thereby reducing the severity of postoperative pain [8,15,16].

**Figure 1:**
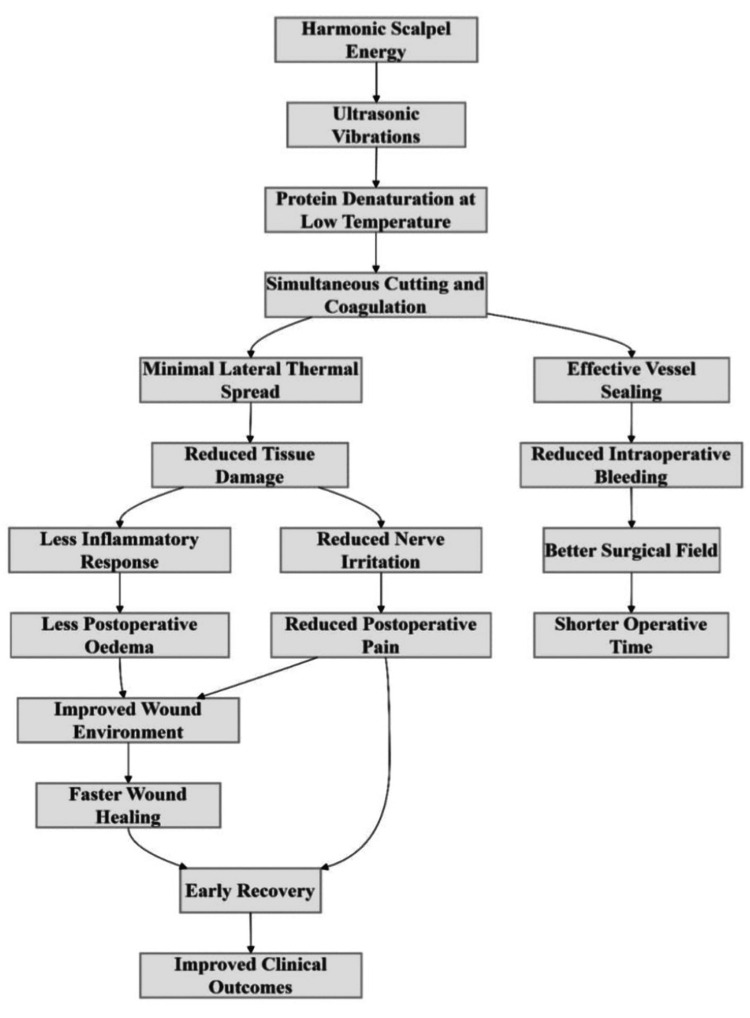
Flowchart illustrating the mechanisms of improved clinical outcomes following hemorrhoidectomy using the HS HS: harmonic scalpel

The mechanism of improved clinical outcomes with HS hemorrhoidectomy is depicted in Figure 1.

Multiple clinical studies have compared the outcomes of the HS technique with the conventional technique. A prospective randomized controlled trial involving 100 patients with grade III and IV hemorrhoids demonstrated lower operating time (7.68 + 2.11 versus 39.12 + 2.37 minutes), postoperative pain scores at 24 hours (1.92 + 0.600 versus 5.54 +0.862), and the duration of hospital stay (1.82 + 0.62 versus 3.04 + 0.66) than the conventional group (p < 0.00001) [8]. Another study conducted among 356 patients with hemorrhoids showed significantly lower operating time (25.55 ± 3.5 versus 36.5 ± 5.1 minutes); lower postoperative pain scores at 12, 24, and 48 hours (<0.0001); shorter hospital stays (1.35 ± 0.4 versus 1.95 ± 0.45 days), fewer complaints of constipation, and lower recurrence rates (8% versus 14%) [17]. A meta-analysis demonstrated that a greater number of patients who underwent hemorrhoidectomy using the HS resumed work within the first postoperative week than the conventional group [18].

In the current study, a statistically significant reduction in operating time (<0.001); intraoperative blood loss (<0.001); postoperative pain on postoperative days 1, 2, and 3 (<0.001); postoperative urinary catheterization (0.018); and the duration of hospital stay (<0.001) was observed in patients undergoing hemorrhoidectomy using the HS compared with the conventional group. Although a statistically significant difference was not observed, postoperative bleeding was lower among patients undergoing hemorrhoidectomy using the HS (n = 1, 1.9%) than the conventional group (n = 2, 3.4%). The findings of our study demonstrate better patient-reported outcomes following the HS than the conventional technique. Overall, these findings align with and strengthen the results of the existing literature.

Strengths of the study

The use of a verified proforma by the principal investigator enabled the assessment and comparison of clinically relevant outcomes, such as operating time, postoperative blood loss, pain, and hospital stay.

Limitations of the study

This was a single-center study with a relatively small sample size and short follow-up duration, which may limit the generalizability of the findings and the assessment of long-term outcomes. Subjective pain assessment may have introduced observer bias. The involvement of different surgeons may have introduced operator-related bias, and as procedures were performed by different surgeons, operator-related variability in surgical technique and experience may have influenced the outcomes. The non-randomized observational design may have resulted in residual confounding despite comparable baseline characteristics between the study groups. In addition, treatment allocation based primarily on patient affordability introduces the potential for allocation bias. The study did not include multivariable adjustment for potential confounding variables or a cost-effectiveness analysis, despite the higher equipment cost associated with hemorrhoidectomy using the HS.

Future recommendations

Multicenter studies with larger sample sizes and long-term follow-ups are recommended to assess the rates of recurrence and anal stenosis. Comparator groups can be employed to assess the cost-effectiveness and quality of life of patients in different groups.

## Conclusions

The HS appears to be an effective alternative to conventional hemorrhoidectomy in patients with grade III and IV hemorrhoids. In the present study, hemorrhoidectomy using the HS was associated with reduced postoperative pain, lower intraoperative blood loss, and shorter hospital stay, resulting in better patient-centered outcomes and faster recovery. However, given the single-center design and short follow-up duration, these findings should be interpreted with caution and validated in larger, multicenter studies.
